# Compounded metformin 10%/triamcinolone 0.1%/gabapentin 6% for treatment of extragenital lichen sclerosus

**DOI:** 10.1016/j.jdcr.2025.07.007

**Published:** 2025-08-16

**Authors:** Fyona Okundia, Rachel Branham, Tiffany Mayo

**Affiliations:** a Center for Clincial Studies, Houston, Texas; b Brookwood Baptist Health, Birmingham, Alabama; c Department of Dermatology, University of Alabama at Birmingham, Birmingham, Alabama

**Keywords:** extra genital lichen sclerosus, lichen sclerosus et atrophicus, sclerosing disorders, skin of color, topical metformin

## Introduction

Extragenital lichen sclerosus (EGLS) represents a small subset of the chronic inflammatory mucocutaneous disease lichen sclerosus (LS).^1^^,^^2^ Unlike classic LS, which predominantly affects the anogenital region, EGLS lesions typically present on the trunk and proximal extremities, beginning as papules that coalesce into atrophic sclerotic plaques over time, and can be pruritic.^1^ The exact etiology of EGLS remains unknown, though autoimmune factors and genetic predisposition have been considered. First-line treatment options include topical ultra-potent steroids and topical calcineurin inhibitors. Second-line options, such as phototherapy, oral steroids, and methotrexate, are also used, but these treatments tend to be less effective for EGLS compared to LS, making EGLS a challenging condition to manage.^1^^,^^2^

Recent interest has emerged in the use of topical metformin for inflammatory and fibrotic conditions like central centrifugal cicatricial alopecia (CCCA) and keloids due to its anti-inflammatory and antifibrotic effects.^3^, ^4^, ^5^, ^6^ Here, we report a novel case of EGLS in an African American woman who experienced significant improvement in plaques following treatment with compounded metformin 10%, triamcinolone 0.1%, and gabapentin 6% ointment.

## Case report

An 80-year-old African American female with a past medical history of breast cancer (status post right mastectomy and tamoxifen greater than 10 years prior to presentation), multiple sclerosis, systemic lupus, hypertension, and hyperlipidemia presented to the dermatology clinic with a long-standing history of lichen sclerosus. The rash had been present for 7 years, primarily affecting the lower abdomen, bilateral arms, genitals, and breasts. Initial treatment with clobetasol 0.05% ointment twice daily yielded minimal improvement.

Two years later, the patient presented with worsening pruritus. Despite continuing clobetasol 0.05% ointment, the patient experienced persistent symptoms. A punch biopsy of the vulva and left upper arm both confirmed the diagnosis of lichen sclerosus.

**Fig 1:**
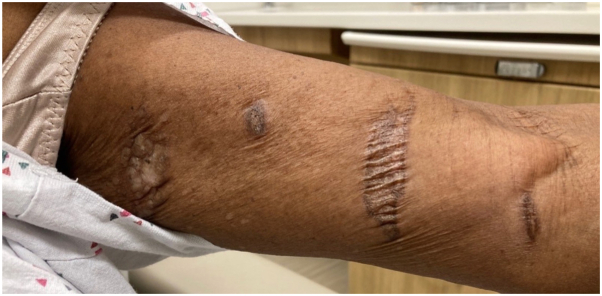
Presentation of left upper arm with scattered hypo- and hyperpigmented atrophic plaques prior to topical metformin 10%/triamcinolone 0.1%/gabapentin 6% compound treatment.

After minimal improvement, tacrolimus 0.1% ointment was initiated, and the patient was started on ultraviolet A1 for extragenital involvement. Despite 11 months of topical therapy and 30 ultraviolet A1 treatments, the patient reported only mild improvement in pruritus but no improvement in the symptomatic flares or overall appearance of lesions (Fig 1).

The patient was then initiated on a topical compounded formulation of metformin 10%/triamcinolone 0.1%/gabapentin 6% with a petrolatum excipient twice daily to extragenital regions and continued on tacrolimus and clobetasol ointment to the vulva. This compounded ointment was trialed on the extragenital region first to ensure tolerability before use on the vulva.

**Fig 2:**
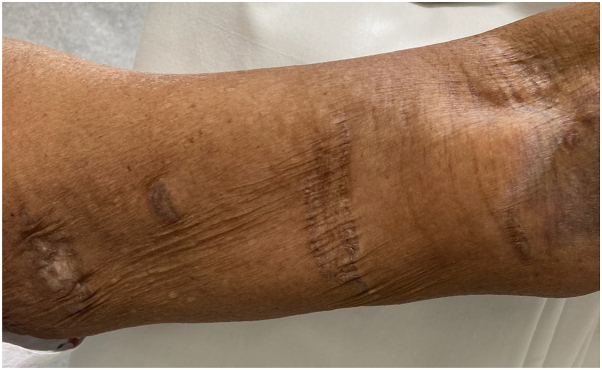
Marked improvement in pigmentation of left upper arm with topical metformin 10%/triamcinolone 0.1%/gabapentin 6% compound after 3 months.

After 3 months of treatment with the compound cream the patient reported improvement in pruritus and significant improvement in the appearance of EGLS. The patient reported no change in the genital region with tacrolimus and clobetasol. On physical examination, the plaques showed marked improvement in texture and pigmentation, particularly on the left arm (Fig 2). Although the patient was happy with the results of her treatment, she discontinued use of the compounded ointment due to its cost, and the vulva was not treated.

## Discussion

Interest in the use of metformin for inflammatory and fibrotic conditions has increased in recent years, following a preclinical trial by Rangarajan et al that demonstrated metformin’s ability to block transforming growth factor-beta and activate adenosine monophosphate activated protein kinase pathways, leading to the reversal of bleomycin-induced lung fibrosis in mice.^5^^,^^7^ Other studies have also shown that oral metformin can clinically suppress inflammation in proinflammatory disease states in patients with systemic sclerosis and hidradenitis suppurativa. Metformin is thought to act as an anti-inflammatory and antifibrotic agent by modulating the transforming growth factor-beta signaling pathway. When this pathway is activated, it phosphorylates transcriptional proteins Sma- and Mad- related proteins (SMAD)2 and SMAD3, which form a complex with SMAD4. This complex translocates to the nucleus, where it promotes the transcription of profibrotic genes, including collagen type 1 alpha 1 chain and collagen type 3 alpha 1 chain. Additionally, metformin activates adenosine monophosphate activated protein kinase, which can inhibit the phosphorylation of SMAD3, thereby downregulating the transcription of profibrotic genes.^8^, ^9^, ^10^

Topical metformin has recently emerged in the literature as a treatment for CCCA. Araoye et al reported the use of topical metformin to stimulate hair regrowth in 2 patients with advanced-stage CCCA who were refractory to treatment with topical minoxidil and intralesional triamcinolone acetonide.^4^ Subsequently, Granja et al reported similar findings in a case report of a patient with CCCA who experienced hair regrowth after topical metformin, despite showing no improvement with topical clobetasol and topical minoxidil.^6^

New clinical studies have reported benefits from using topical metformin for fibrotic skin conditions such as keloids and hypertrophic scars. A blinded clinical trial involving 53 participants examined the effects of topical metformin ointment on these conditions. The study found that, over 3 months, topical metformin significantly reduced scar height, vascularity, pigmentation, and pliability compared to placebo.^3^

This compound contains medium potency triamcinolone 0.1% which could aid in EGLS symptomatology; however, the patient was previously minimally responsive to super-high potency clobetasol 0.05%. Gabapentin 6% could have played a role in decreased sensation of pruritus. The metformin 10% was utilized for its anti-inflammatory and antifibrotic properties which we hypothesize may have led to an additive response in combination with triamcinolone 0.1% and gabapentin 6%.

In the case presented, the patient was refractory to standard first- and second-line therapy for EGLS. However, after 3 months of treatment with topical compounded metformin 10% ointment, the patient showed notable improvement in the texture, appearance, and pigmentation of plaques, with no adverse effects. These clinical findings suggest that topical compounded metformin may be a novel and effective treatment option for patients with EGLS and other fibrotic sclerosing skin conditions. Further research is essential to fully determine its efficacy as a treatment for EGLS and other fibrotic sclerosing skin conditions.

## Conflicts of interest

None disclosed.
